# A39 IMPACT OF AN ENDOSCOPIC OPTICAL DIAGNOSIS COURSE ON GASTROINTESTINAL NEOPLASM CHARACTERIZATION SKILLS AMONG TRAINEE ENDOSCOPISTS

**DOI:** 10.1093/jcag/gwae059.039

**Published:** 2025-02-10

**Authors:** W Tran, N Gimpaya, R Khan, C Walsh, L Hookey, M Rai, S Grover, R Bechara

**Affiliations:** Education Research, Scarborough Health Network, Scarborough, ON, Canada; Education Research, Scarborough Health Network, Scarborough, ON, Canada; Division of Gastroenterology, Mayo Clinic Minnesota, Rochester, MN; SickKids Research Institute, Toronto, ON, Canada; Queen’s University, Kingston, ON, Canada; Queen’s University, Kingston, ON, Canada; Education Research, Scarborough Health Network, Scarborough, ON, Canada; Queen’s University, Kingston, ON, Canada

## Abstract

**Background:**

Real-time endoscopic optical diagnosis involves detailed visualization of the digestive mucosal and microvascular pattern, allowing for prediction of histology. This is advantageous as it can guide endoscopic resection decisions, surgical referrals and provide cost savings to histopathology. There is, however, no standardized or widely implemented curriculum for endoscopists or trainees to learn optical diagnosis skills.

**Aims:**

To evaluate the impact of a course for the optical diagnosis of esophageal squamous cell carcinoma (ESCC), early gastric cancer (EGC), Barrett’s esophagus (BE), and colorectal polyps

**Methods:**

19 gastroenterology trainees were invited to the 2-day course in Kingston, Canada. Number of procedures previously performed was recorded. The impact of the course on lesion diagnosis accuracy was evaluated using a pre-test, immediate post-test, and delayed post-test administered 6 weeks after the course. A repeated measures ANOVA was performed to compare the mean score (%) between the three tests.

**Results:**

13 trainees completed all the tests (Table 1). Mean score % values for the overall tests and test scores for esophageal, gastric, and colon lesions were summarized on Table 1. The repeated measures ANOVA determined that the mean score % had a statistically significant difference between the three tests (F (2,24)=5.63, P=0.01). Pairwise comparisons revealed that there was a statistically significant increase in mean scores between pre- and post-tests (13.86 (95% confidence interval (CI) of 4.36 to 23.33), p<0.01) with no significant difference between post and delayed post-tests (8.46 (95% CI of -0.12 to 17.04), p>0.05) (Figure 1). The mean correct % from the delayed post-test was higher than the pre-test but there was no statistical significance (5.39 (95% CI of -3.72 to 14.49), p>0.05).

**Conclusions:**

This novel optical diagnosis course improved trainee accuracy in diagnosing ESCC, EGC, BE and colorectal polyps. Learning retention was evident, with no significant score difference between immediate and delayed post-tests. This study was limited by the low sample size and low number of test items. Further investigation is required to validate the course for international trainees and explore its clinical transferability.

Demography and Mean Score %

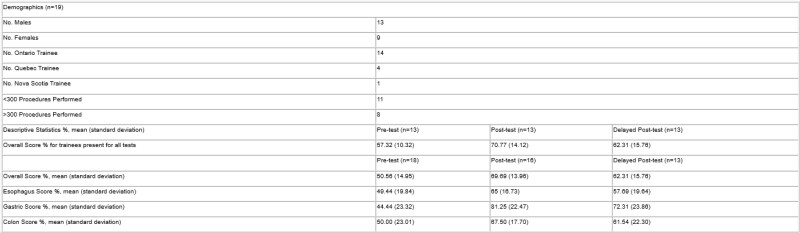

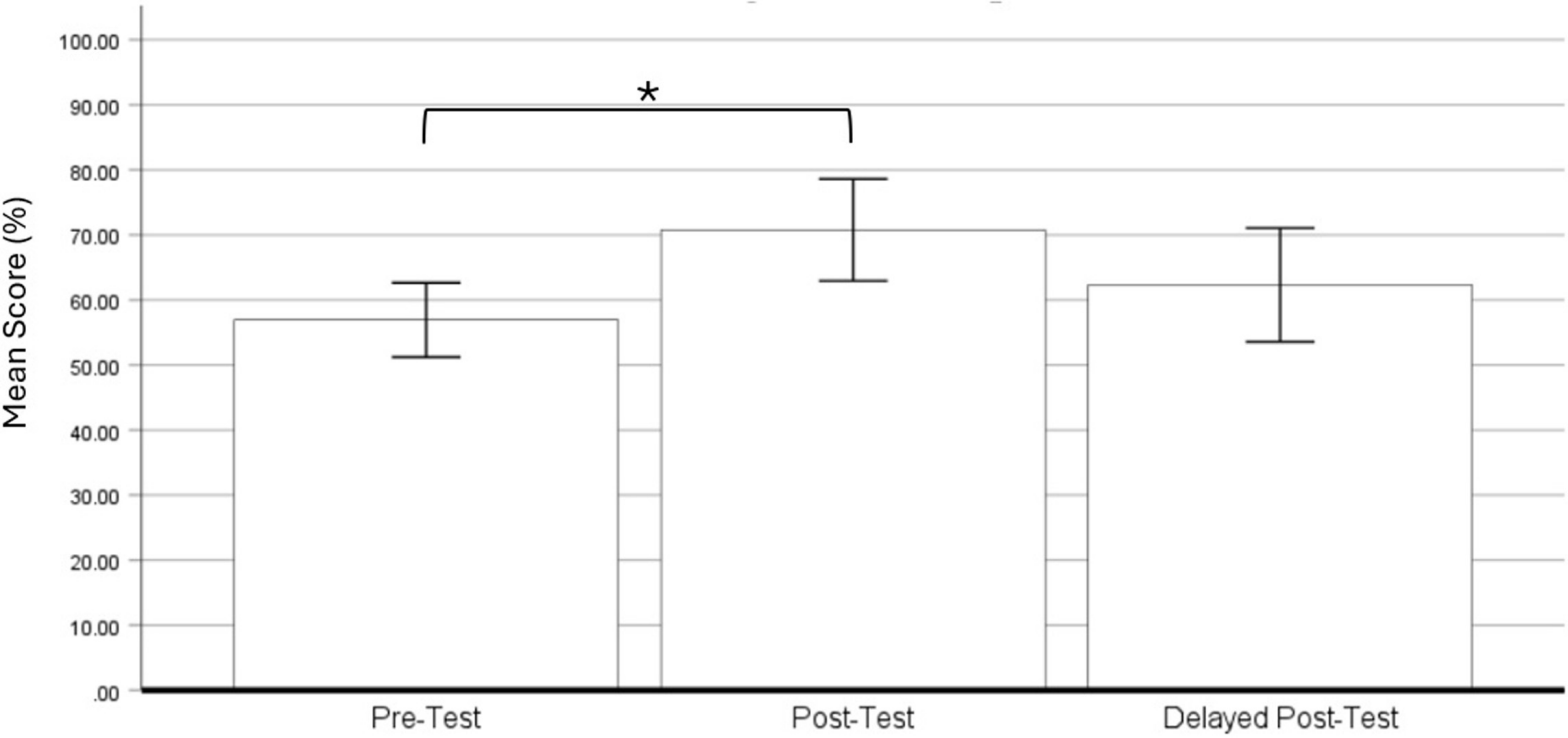

Figure 1. Mean score %. (*) denotes p<0.05.

**Funding Agencies:**

None

